# Addressing the links between and internal vs. external regulation factors, achievement emotions and gender in problematic use of ICT at university

**DOI:** 10.3389/fpsyg.2024.1382016

**Published:** 2024-08-06

**Authors:** Jesús de la Fuente, Leyre Lecuona-López, Mónica Pachón-Basallo, Leyre San Martín-Íñiguez, Pablo Blanco-Sarto

**Affiliations:** ^1^Department of Psychology, School of Education and Psychology, University of Navarra, Pamplona, Spain; ^2^Department of Systematic Theology, School of Theology, University of Navarra, Pamplona, Spain

**Keywords:** internal vs. external regulation behavior, achievement emotions in class, gender, university, ICT

## Abstract

**Context:**

The study of internal and external regulation in the use of Information and Communications Technology (ICT) and the analysis of academic emotions have become increasingly important due to their impact on academic life at university.

**Objectives:**

This research aims to investigate the links between internal vs. external regulation factors, achievement emotions, and gender in the problematic use of ICT among university students.

**Methodology:**

The study recruited 317 university students, predominantly female (72.51%), using an ex-post facto design. The SRT-ERT scale was used to assess technology-related behaviors, and the AEQ Scales measured academic emotions before, during, and after class. Correlation analysis, multiple regression analysis, and ANOVA were employed to identify significant relationships.

**Results:**

Significant relationships were identified between regulatory behaviors, with male students exhibiting more dysregulated behavior and greater exposure to dysregulatory technology environments. Individual and contextual behavioral nonregulation and dysregulation in the use of ICT were significantly predictive of negative academic emotions.

**Conclusion:**

The findings suggest that both personal and contextual factors of regulation significantly influence the emotional experiences of students in academic settings. The study highlights the need for psycho-educational interventions to promote better regulatory behaviors among university students, particularly males.

## 1 Introduction

### 1.1 Issues in the use and abuse of ICT in university students

Over recent years, ICT has become an increasingly important part of our daily lives. The COVID-19 pandemic significantly changed how we work, study and relate to other people with a radical shift to the use of various devices (Ariza et al., 2021; Gea et al., 2021). UNESCO (2021) reports an increase in the use of ICT in education but highlights that 43% of young people lack basic digital skills, limiting their ability to take advantage of the opportunities presented by ICT. In Spain, 94.5% of people use the Internet every day, principally young people aged 16–24 (Instituto Nacional de Estadística, 2023). That increase can lead to dependence and screen addiction, with possible adverse effects on the mental and physical health of young people, particularly university students (aged 18–22; Gea et al., 2021).

Psychological factors such as loneliness, impulsiveness and reduced parental supervision in this life stage can lead to greater use of the Internet (Li et al., 2021). The inappropriate use of ICT can affect both physical and mental wellbeing in students and can affect the immune system (Shields et al., 2017; Li et al., 2021), which presents concerning risks for their development (UNESCO, 2021). Prior research has identified adverse effects, including anxiety, sleep disorders, depression, poor mental health and poor academic achievement (Alimoradi et al., 2019; Wong et al., 2020; Sanders et al., 2023), associated with nomophobia (fear or anxiety at not having your phone) and addiction to mobile devices (Díaz-Miranda et al., 2020).

A significant relationship has also been found between inappropriate use of the Internet and poor academic achievement (Aznar-Díaz et al., 2020), particularly in students who are repeating a school year, where a direct relationship has been observed between ICT abuse and poor achievement (Barrio, 2018). ICT has been found to be the principal distraction from the teaching and learning process (Díaz-Vicario et al., 2019; De la Hoz, 2021). In particular, frequency of use of videogames is inversely related with academic achievement and has been associated with a lack of motivation for academic tasks (Díaz-López et al., 2021). Conversely, it is notable that responsible use of ICT is positively associated with better academic achievement (Díaz-López et al., 2021; Sanders et al., 2023). It is crucial to be vigilant as to how ICT is being used in order to minimise its negative impact on academic achievement and the mental health of students.

### 1.2 Conceptualisation of the use and abuse of ICT

Using ICT properly is essential in the twenty-first century, particularly for young people. Young people often lack the tools to regulate their use of ICT and are thus exposed to a number of risks (Sánchez-Caballé et al., 2020). Consequently, well-adjusted, responsible use of ICT should be promoted so as to allow young people to enjoy its benefits for learning, communication and work whilst ensuring their safety and building a positive digital society. This focus entails adjusting the use of ICT to the needs of young people and giving them critical, ethical skills and stressing the need to respect moral principles and values in order to avoid the risks of inappropriate use (Prats-Fernández et al., 2018; Moreira-Sánchez, 2019).

Digital competence is an important element of current education and comprises a variety of skills, including management of information, content creation, communication, ethical skills, problem-solving, technical and strategic skills. Terms such as ‘digital literacy’ and ‘digital competence’ are often used to refer to this set of complex learnings (Sánchez-Caballé et al., 2020).

Some people mistakenly believe that young people have these skills innately, but most experts do not believe that they do in fact have all the digital skills required (Moreira-Sánchez, 2019; Sánchez-Caballé et al., 2020). As such, educational institutions have a fundamental role in helping students to develop this competence. It has been proposed that digital competence should be part of the university curriculum with a focus on the development of critical digital and ethical skills (Gómez-Vahos et al., 2019).

However, addiction to mobile devices is not recognised as a specific disorder in the Diagnostic and Statistical Manual of Mental Disorders (DSM-5). The DSM explains that there is as yet insufficient evidence to include Internet Gaming Disorder as an official diagnosis and as such it falls under Section 3, ‘Conditions requiring further study’. A series of criteria are put forward and further study is recommended. Nevertheless, the World Health Organisation, in the most recent edition of its manual (ICD-11; World Health Organization, 2019) includes in its chapter on disorders of addiction ‘Gambling Addiction’ (online and offline) and ‘Videogame Addiction’. The scientific literature lacks consensus on how to categorize such behaviors, which are variably treated as behavioral addictions, mobile abuse, or evident risk factors (Salehan and Negahban, 2013; Díaz-Miranda et al., 2020; Laurence et al., 2020; González-Cabrera et al., 2022; Chou et al., 2024).

Problematic use of ICT can be seen as an inability to control the use of cyberspace, with unease when not using. or when deprived of access to, technology (Díaz-López et al., 2021). Problematic ICT use can also negatively impact individuals’ day-to-day life, who might fail to meet social, educational or family commitments, and spend excessive time using ICT (Baloğlu et al., 2020).

### 1.3 Protective and risk factors: explanatory models

#### 1.3.1 Protection model: behavioral competence

*Protective factors* are characteristics of the individual, family, group or community that favour human development and wellbeing. Those factors reduce vulnerability in general or in specific ways and help to mitigate the effects of risk factors (de los Páramo, 2011).

Competence-based education (CBE) is a model of education that focuses on the development of practical, transferable abilities, skills and knowledge in order to handle real-life situations and solve problems effectively (Irigoyen et al., 2011). The concept of competence entails the ability or skill to perform a tasks effectively in a given context (Tobar-Otero and Canós-Darós, 2022). The *competence-based model* has three dimensions (Gagné, 1965, 1967; de la Fuente, 2023a,b):

1. *Conceptual* knowledge or Knowing how to know. Refers to understanding and using concepts, theories and principles in a given field of knowledge, showing deep knowledge of fundamental concepts.
2. *Procedural* knowledge or Know-how. This is the capability (skill and meta-skill) to apply knowledge and skills in order to carry out a task successfully and effectively. Knowing how use skills in real life. The most notable aspects of procedural knowledge are meta-cognitive skills, which include strategies for the control and regulation of the learning process (Callejas, 2015). Such skills include self-regulation of learning, planning, monitoring, critical evaluation of information, self-knowledge and reflection on thinking and learning (de la Fuente and Justicia, 2018). Self-regulation is crucial to the control and regulation of the learning process (Romo-Sabugal et al., 2020) and the use of technology.
3. *Attitudinal* knowledge or Knowing How to Be. This comprises the attitudes, values, motivation and emotions required for adequate performance in social and employment contexts (Callejas, 2015; López-Gómez, 2016).

#### 1.3.2 Risk-protection models focused on personal characteristics: self-regulation

Albert Bandura’s Cognitive-Social Theory underlines the importance of self-efficacy for motivation and behavior. Performance or achievement in an activity depends on perceived self-efficacy, the skills required and incentives (Bandura, 1991). The theory suggests that self-efficacy regulates behavioral, affective, motivational and cognitive processes and decision-making (Bandura, 1986). Students with high expectations of self-efficacy have greater motivation, better academic results and show greater self-regulation and intrinsic motivation to learn. Self-efficacy affects perseverance and motivation through goal setting (de la Fuente et al., 2022c). Social skills and a sense of belonging are positively associated (Liu et al., 2020). Behavioral self-regulation, such as planning, monitoring and evaluation of the subject’s behavior, acts as a protective factor (de la Fuente et al., 2022c; de la Fuente, 2023a,b) and allows the subject to be active in pursuit of their long-term objectives (Del Castillo and Días, 2007). According to Zimmerman’s Theory of Self-Regulation, self-regulation is positively associated with personal adjustment and academic achievement (de la Fuente et al., 2022c). These factors protect the academic success and psychological wellbeing of students (Li et al., 2021).

There are factors in adolescence that can increase the likelihood of young people adopting risk behaviors and developing disorders common to this stage of life, whether social, family or personal. Such factors are said to be risk factors because they increase the vulnerability of adolescents to such issues (de los Páramo, 2011). Personal risk factors include depression, social isolation, anxiety, low self-esteem, feelings of loneliness and worthlessness, hopelessness and poor self-efficacy in coping with conflict and handling negative emotions (Gómez-Tabares et al., 2019). Examples of risk factors include academic stress, lack of social support, anxiety and depression (de la Fuente et al., 2019). There is also a negative relationship with personality traits such as scrupulousness (de la Fuente et al., 2022c).

Alongside that, emotional intelligence (EI), self-regulation and resilience are personal resources that positively impact the psychological wellbeing of adolescents (Del Castillo and Días, 2007; Chamizo-Nieto and Rey, 2020). High levels of emotional and social skills, associated with emotional management and interpersonal interactions, are themselves associated with lesser aggression and antisocial behavior (Nasaescu et al., 2018). Developing emotional skills such as identification, comprehension and emotional regulation facilitates adaptive coping with stress in daily life, so increasing subjective wellbeing (Chamizo-Nieto and Rey, 2020).

Conversely, impulsiveness and lack of psychological resources are associated with the risk of suicide in young people (Gómez-Tabares et al., 2019) and Internet addition, as a result of problems with inhibition control and emotional regulation (Gómez-Tabares et al., 2019; Li et al., 2021). A person’s low perception of their own social skills can favour the development of a preference for online interaction and increase the risk of ICT abuse (Nasaescu et al., 2018). In addition, evidence shows that a deficit of emotional regulation is a risk factor for cyberaggression (Chamizo-Nieto and Rey, 2020).

#### 1.3.3 Risk-protection models focused on Context

In relation to context, there are associated risk factors that may impact the mental health of students, such as being removed from or distant social and family support networks, dysfunctional parenting and situations of psychosocial and academic stress (Gómez-Tabares et al., 2019). Albert Bandura indicated in the *Theory of Learning from Observation* that children’s social models are inseparable from their context, since they learn, imitate and recreate behavior from their most immediate environments, such as family and school (Rodríguez-Rey and Cantero-García, 2020). It has been seen that the imprisonment of their father has lasting negative effects on the mental and social wellbeing of children, particularly those from ethnic minorities. Those effects may manifest in emotional behavioral problems and poor social adjustment (Del Toro et al., 2023).

People who have grown up in a dysfunctional family environment tend to develop behavioral disorders at a young age. Such an environment is a risk factor for antisocial behavior, offending and increased negative emotionality in children (Vera-Sánchez and Alay-Giler, 2021; Balladares-Fiallos and del Ponce-Delgado, 2022). It has also been found that the attitude of parents to the use of the Internet impacts the possible negative consequences on children (Pan et al., 2020). Several authors have shown that the family is very important for the development and wellbeing of future generations and plays a fundamental role in the formation of values, standards and habits (Ariza et al., 2021; Bernal et al., 2021; Balladares-Fiallos and del Ponce-Delgado, 2022).

The educational context may impact the health of students (van den Toren et al., 2020). In that regard, it has been observed that there is a close connection between self-efficacy, confidence in ones own performance, and self-regulation. When that relationship is positive, there is a greater feeling of control over our actions and their outcome. As such, we can understand that students with learning difficulties tend to have lower self-efficacy and stronger focus on external factors than on personal aspects. It has also been observed that a feeling of belonging to the educational setting predicts academic achievement. And associated with that, we find two significant contextual factors: school support for learning and acceptance of diversity (Liu et al., 2020).

#### 1.3.4 Interactive protection-risk models: SR-ER behavior

The *Theory of Self vs External Behavioral Regulation* (SR vs. ER, de la Fuente, 2017, 2021; de la Fuente et al., 2022c) and the *Conceptual Utility Model* (de la Fuente and Martínez-Vicente, 2023a,b, 2024) have postulated a continuum of the personal and contextual variable of regulation. Human behavior has to be understood as a whole, as a combination of personal and contextual variables. Thus, the model is interactive and intended to determine the relationship between an individual’s personal characteristics and their context (de la Fuente, 2017; de la Fuente et al., 2022c) as a mechanism to explain the technology-related behavior of individuals.

The model has three core principles: a person’s level of behavioral regulation, the regulatory characteristics of the context and the interaction or combination of personal characteristics and context. Under the model, there are presage or predictive factors of personality and context that explain in combination the behavioral variability under analysis:

1. Individual factors: individuals’ levels of self-regulation in relation to ICT, in the sense of their capacity to manage their own behavior and emotions, can be categorised as follows: *High*: Proactive/Self-Regulation; *Moderate*: Non-regulation/Reactiveness or non-regulatory behavior (NR); *Low*: Dysregulation (DR). Moreover, self-regulation is crucial in determining appropriate technology use and offers protection against addictive behaviors. Research shows that behavioral self-regulation (impulsiveness and lack of control) significantly impacts where individuals fall on the continuum of appropriate to abusive technology use (Javaeed et al., 2019; Saad and Gamal, 2020). The term “behavioral addiction” highlights the maladjustment due to lack of self-regulation in using technological devices (Kuss et al., 2014; Zhao et al., 2024).
2. *Contextual factors*: The environment affects regulation of ICT to the extent that it offers environmental stimuli that predispose to and impact the behavior of the individual, whether in favour of or so as to hinder self-regulation (de la Fuente, 2017; Pachón-Basallo et al., 2021). The *Theory of Self vs External Behavioral Regulation* postulates the following levels: *High external regulation:* Regulatory Context (ER); *Medium level of external regulation:* Non-regulatory Context (ENR); *Low external regulation*: Dysregulatory Context (EDR). Contextual factors are predictive of technology-related addictive behaviors (Li et al., 2021). However, the interaction between an individual’s self-regulation level (SR-NR-DR) and their context (ER-ENR-ED) remains underexplored. Understanding this interaction can advance our knowledge of the factors contributing to addictive vs. non-addictive technology use. The different levels predicted by this theoretical model need further empirical validation.
3. The SR vs. ER Theory predicts that a student’s self-regulation and the regulatory nature of the context jointly determine motivational-affective variables. This principle has been tested, showing that self-regulation levels (low-medium-high) and the regulatory nature of teaching (low-medium-high) determine positive or negative emotions and burnout/engagement levels. These findings support the use of a five-level progressive scale to understand student-teacher interactions and their impact on emotional health. The theory’s constructs (SR-NR-DR; ER-ENR-EDR) help understand learning, teaching, and achievement factors by considering student and context characteristics. This understanding aids in developing psychoeducational intervention strategies. In health and educational psychology; these constructs help profile individuals’ self-care practices, promoting health and understanding behavioral contexts that support or hinder health (de la Fuente et al., 2022c).

### 1.4 Academic emotions in class

Academic emotions span the spectrum of emotions experienced in connection with academic activities, assessment and achievement involved in the teaching and learning process. Academic emotions are chiefly categorised along two dimensions: valence (positive or negative) and activation. *Positive emotions*, such as enjoyment, hope and pride are associated with gratifying, stimulating academic experience whilst negative emotions include emotions that activate such as anxiety and anger and emotions that deactivate, such as boredom and hopelessness (Pekrun et al., 2007; Pekrun, 2017).

Academic emotions have a significant impact on achievement and academic adjustment among students. *Positive emotions* such as enjoyment, pride and hope have been shown to be positively predictive of better grades for both university and secondary school students (Barron and Harackiewicz, 2001; Pekrun et al., 2004, 2009; Frenzel et al., 2007; Goetz et al., 2008). Conversely, deactivating negative emotions such as boredom and hopelessness have consistently been shown to be negatively correlated with academic achievement (Pekrun et al., 2004, 2009; González et al., 2009).

A recent study by examined how *perfectionism* dimensions and positive/negative affect relate to perceived motor competence in Spanish primary school children aged 8–12 years. They found that all dimensions of perceived motor competence (physical conditioning, sports competence, strength competence, self-confidence, peers and self-experience) were positively correlated with self-oriented perfectionism or the tendency toward self-improvement. This highlights how positive perfectionism tendencies can influence self-perceptions of ability in the physical domain during childhood Additionally, a study by, examined the self-perceived affective states of primary school students in Physical Education classes, considering gender and body mass index (BMI). The findings revealed that students without obesity/overweight issues reported higher positive affect scores, while those with obesity/overweight problems had higher negative affect scores. These results underscore the importance of addressing emotional well-being in educational settings to promote positive affective experiences and mitigate negative ones, particularly in relation to physical health and body image.

In relation to ICT use, the systematic review carried out by Wu and Yu (2022) which examined 23 publications concluded that *positive emotions* such as enjoyment, pride and relaxation tend to positively impact on different aspects of online learning, such as motivation, performance, satisfaction and achievement. However, it has been challenging to clearly describe the effects of *negative emotions* on the outcome of online learning given the divergence of views about their effects. It is important to highlight that both positive and negative academic emotions influence fundamental aspects of the educational process, including effort, learning strategies and, finally, academic achievement (Pekrun et al., 2004, 2007; Pekrun, 2017). As such, understanding the role of emotions in education is crucial not only in order to improve students’ experience of learning and performance, but also so as to analyse the role of emotion in the use of ICT in educational environments.

### 1.5 The influence of gender

Prior research looking at gender as a mediating or modulating factor has shown that both genders spend more time online for leisure than for study or work (Fernández-Villa et al., 2015). There are, however, differences in the time spent online by each gender, albeit they are both at the same risk of becoming dependant or abusing the internet (Salehan and Negahban, 2013; Gea et al., 2021).

First, *males* show greater self-efficacy (in the sense of a judgement of a person’s own capacities) and a more positive attitude towards the use of ICT (Padilla-Carmona et al., 2022). Men who have higher levels of restriction have a lesser likelihood of becoming addicted to the Internet (Li et al., 2021). However, it has been found that being *male* is a significant predictor of the development of Problematic Internet Use (PIU). Men are more vulnerable to the most severe symptoms of PIU and the condition has greater prevalence among them (Baloğlu et al., 2020; Díaz-López et al., 2021).

Conversely, females have been seen to place greater value on things published online and are more influenced by online content (Li et al., 2021). The use of the internet among females is more closely associated with their social and emotional environments. In addition, statistically significant differences have been found in time management, with perceived negative impacts on personal and social life due to the time devoted to Internet use (Baz-Rodríguez et al., 2020).

Moreover, gender differences in ICT skills have been observed. A study on Finnish upper comprehensive school students found that boys tend to score better on technical-oriented items, while girls excel in school work-oriented and social interaction-related items (Kaarakainen et al., 2017). This suggests that gender differences in ICT skills are more item-specific than general.

Gender also influences online preferences. Women tend to spend more time on online shopping, social media and chatting, for social reasons, whilst men prefer online betting and videogames and use the internet for recreation (Fernández-Villa et al., 2015; Andreassen et al., 2016; Yudes et al., 2019; Gea et al., 2021). The gender factor moderates PIU, associated with anxiety in women, and more widespread problematic videogaming in men (Baloğlu et al., 2020). Personal factors such as hyperactivity, impulsivity and poor attention also predict problematic use of mobile devices (Laurence et al., 2020).

Additionally, gender differences in internet addiction have been studied. Research indicates that both genders share social media addiction as the primary predictor of internet addiction, but there are exclusive predictors for each gender. This highlights the importance of considering gender-specific variables in the development and treatment of internet addiction (Mari et al., 2023).

Although videogame disorder has historically been more associated with men (Pan et al., 2020), the videogame industry has concentrated on men, contributing to higher use among men (Castro et al., 2015). Whilst gender may affect attitudes towards the use of the internet, there is no consensus as whether it is a determinative variable (Gea et al., 2021).

Finally, gender also has a role in academic emotions. It has been found that there is a significant gender-based difference in positive emotions associated with the academic environment. Women tend to score more highly for emotions such as enjoyment, hope, and pride relative to men (Barron and Harackiewicz, 2001; Pekrun et al., 2004, 2007). However, gender differences are not so clear or consistent for negative emotions. Understanding student anxiety is crucial for broadening gender diversity in STEM fields (Science, Technology, Engineering, and Mathematics), as it disproportionately and negatively influences women. Research has shown that women may be more likely to become trapped in a self-deprecating cycle driven by negative academic emotions, which can affect their retention in STEM (Pelch, 2018).

### 1.6 The importance of preventive intervention

Use of the internet has increased significantly over recent years. Currently, 99.1% of students use the internet every day (Garrote-Rojas et al., 2018), which has led to excessive use among youth populations (Díaz-Miranda et al., 2020). Young people are the population at greatest risk of PIU and over recent years, Spain has recorded one of the highest levels of PIU among adolescents in Europe (Aznar-Díaz et al., 2020). Inappropriate use of ICT is a significant problem among young people, with evident consequences (Kokka et al., 2021). Since the phenomenon has many causes, from a biopsychosocial perspective, it is key to identify the determining factors that affect inappropriate use of ICT.

Providing education about appropriate use of ICT is fundamental to the prevention of problems at personal, social, academic and family contexts. The analysis should be approached from the perspectives of students, teachers, counsellors, family members and experts (Díaz-Vicario et al., 2019). That would enable a practising general psychologist to offer strategies to strengthen a person’s internal regulation and the regulatory effect of their family and educational environment.

A comprehensive review conducted by Muñoz-Oliver et al. (2022) highlights the importance of integrating emotional intelligence (EI) into the curriculum to enhance students’ socio-emotional competencies. The study reviewed 41 emotional education programs implemented in educational settings over the last decade and found that these programs significantly improve students’ emotional regulation, social skills, and overall academic performance. The review also emphasized the need for teacher training in socio-emotional competencies and the involvement of families in program interventions. This aligns with the broader understanding that both positive and negative academic emotions influence fundamental aspects of the educational process, including effort, learning strategies, and, finally, academic achievement (Pekrun et al., 2004, 2007; Pekrun, 2017).

Psychologists in general practice working with educational psychologists have a key role in *primary prevention*. They can set up Family Schools (Spanish: Escuelas de Familia; de la Fuente, 2023b) to provide support in early affective relationships, bodily changes, early exposure to substances of abuse and the use of technology (Echeburúa et al., 2012). Such Schools can be a valuable tool to help adolescents who are both victims and perpetrators of cyberbullying by giving them appropriate tools (Quintana-Orts et al., 2023).

The focus of *secondary prevention* is direct work with young people. The aim is to teach coping strategies to handle online conflict, promote self-efficacy and boost the socio-emotional competencies of adolescents and young adults (Divecha and Brackett, 2020; Quintana-Orts et al., 2023). Interventions are focused on personal protective and contextual factors so as to maximise the internal capacities of adolescents, such as social skills, which are essential to their protection (Liu et al., 2020; van den Toren et al., 2020).

### 1.7 Objectives and hypothesis

In light of the aforementioned issues, this study examines the behavior of university students as users of information and communication technologies (ICT). The aim is to identify the internal and external factors that predict such behavioral patterns and their relationship with positive and negative emotions among university students. The specific objectives were:

1. To identify the levels of more and less frequent regulatory factors.
2. To determine the associations and predictive relationships between different levels of regulation, whether personal or contextual, and achievement emotions in class.
3. To analyze the influence of external regulation on internal regulation and to assess the impact of gender on the identified differences.

On the basis of those objectives, the following *hypotheses* were tested:

#### 1.7.1 Descriptive hypotheses

1. The level of *Internal and External Regulation of Technology* (SRT, ERT) will be greater than internal and external Non-Regulation of Technology (NRT, ENRT) and internal and external Dysregulation of Technology (DRT, EDRT). In parallel, the level of SRT will be greater than the level of ERT.
2. The level of positive academic achievement emotions (enjoyment, satisfaction, pride) will be greater than negative emotions (boredom, anxiety, anger, hopelessness) in class. Among negative emotions, *boredom* will be significantly higher.

#### 1.7.2 Association and linear prediction hypothesis

1. *Levels of External Regulation of Technology* (ERT/ENRT/EDRT) will show associations that are significant and positive with levels of Self-Regulation of Technology (SRT/NRT/DRT). It is assumed that *Self-Regulation of ICT* (SRT) will be negatively correlated with and negatively predictive of NRT, and ENRT, and DRT and EDRT.
2. It was hypothesised that *Internal and External Regulation of ICT* (SRT, ERT) will show positive correlations with and be positively predictive of positive emotions. However, NRT and ENRT and specially DRT and EDRT will show positive correlations with and be positively predictive of negative emotions. It is expected that this effect will be especially significant with *boredom* during class.

#### 1.7.3 Inferential hypotheses

1. *Levels of External Regulation of Technology* (ERT/ENRT/EDRT) will determine *levels of (internal) Self-Regulation of Technology (SRT/NRT/DRT).*
2. The *average score for SRT, NRT and DRT* will determine the highest level of positive emotions and the lowest (or worst) level of negative emotions in class. It is expected that these variables will have a more material impact on the prediction of academic emotions in comparison with external regulatory variables (*ERT, ENRT, EDRT*).
3. *Gender* will significantly determine differences in levels of SRT and ERT among university students. In fact, *males* will score higher, and that difference will be significant, for DRT and EDRT. In relation to academic emotions, it is expected that *male* students will experience negative emotions to a greater extent than *female* students.

## 2 Materials and methods

### 2.1 Participants

A total of 317 university students at different universities participated in the study. The study was conducted in the R&D framework of the Red Inetas^1^ and the specific R&D projects in question. Students were invited to participate in the study voluntarily. Of the participants, 230 were female (72.51%) and 87 were male (27.49%). Ages were in the range 18–25 (m = 20.08, SD = 2.37). The sample comprised students enrolled in social sciences courses, all of whom participated voluntarily. Due to the impossibility of creating a random sample, an incidental, non-probabilistic sample was used. An incidental, non-probabilistic sample refers to a sample selected based on convenience, without following a random or probability-based selection process. The inclusion criterion for the study was being a university student. The exclusion criterion was the absence of prior clinical pathologies or diagnoses recorded in the self-reported consent form.

### 2.2 Instruments

#### 2.2.1 Self-regulatory behavior and contextual regulation

SRT and ERT were evaluated using the Scale for the Evaluation of the Use of Technological Devices in Class at University (SRT-ERT; de la Fuente, 2022, 2024a,b). This scale contains 36 item answered on a Likert scale (1 = Totally disagree; 5 = Totally agree) which in turn each contain six susbscales: SRT (“I consider the appropriate use of ICT in class”); NRT (“I seldom consider the appropriate use of ICT in class”); DRT (“I take decisions to be able to use ICT in class how I want, because nobody can tell me how to use ICT.”) ERT (“The environment in class (university, teachers other students) helps me to plan my use of ICT in class by setting goals and targets”); ENRT (“In the environment in class (university, teachers other students) people rarely discuss appropriate use of ICT.”); and EDRT (“The environment in class (university, teachers other students) helps me to really enjoy using ICT, because they encourage me to do what I want, if it makes me happy.”). The factorial structure showed acceptable values [Chi square = 1,628,730, *p* < 0.001; df (702–118) = 584; CH/DF = 2,789; CFI = 0.927; GFI = 0.903; IFI = 0.926; TLI = 0.926; RMSEA = 0.023; RSMR = 0.042; Hoelter = 1,309 (*p* < 0.05), 1.360 (*p* < 0.01)) The total reliability of the scale was also satisfactory (α = 0.916; ω = 0.885). The subscales also demonstrated acceptable reliability: SRT (α = 0.881; Omega = 0.876); NRT (α = 0.701; ω = 0.683); DRT (α = 0.858; ω = 0.834); ERT (α = 0.943; ω = 0.925); ENT (α = 0.865; ω = 0.850); EDT (α = 0.915; ω = 0.901).

#### 2.2.2 Academic emotions

The *Achievement Emotions Questionnaire* (AEQ), (Pekrun et al., 2005, Spanish language version Paoloni, 2018), is a multidimensional self-reported questionnaire for the assessment of achievement emotions in university students before, during and after class. It has 80 Likert items that measure four *positive emotions* (enjoyment, hope, pride, relief) and five *negative emotions* (anger, anxiety, hopelessness, shame and boredom) through items such as “I am ashamed that other people understand the class material better than me” and “During class, I want to vanish and disappear from my seat.” The scale has demonstrated validity and reliability with a consistent, acceptable factorial structure in this sample. The confirmatory model presented good fit [Chi-square = 529.890; Degrees of freedom = 79; Ch/df = 6.70; SRMR = 0.053; *p* > 0.08; NFI = 0.964; RFI = 0.957; IFI = 0.973; TLI = 0.978, CFI = 0.971; RMSEA = 0.080; HOELTER = 165 (p < 0.05) and 178 (p < 0.01)]. Good internal consistency was also found for the total scale (Alpha = 0.939; Part 1 = 0.880, Part 2 = 0.864; Spearman-Brown = 0.913 and 884; Guttman = 0.903). Example items include: Item 90: I get angry when I have to study; Item 113: My sense of confidence motivates me; Item 144: I’m proud of myself.

#### 2.2.3 Procedure

Students gave written informed consent and subsequently completed the instruments online anonymously. The R&D Project was approved by the Research Ethics Committee of the University of Navarra (Ref. 2018.170) and University of Almería (Ref. UALBIO2024/050). Students were invited to participate using an anonymized QR code. After reading the Participant Information Sheet and the Self-Informed Consent Sheet, they were invited to complete different inventories through the inetas online Platform (www.inetas.net; de la Fuente et al., 2015). They were asked to complete a seminal inventory during a quarter. The SRT-ERT inventory was completed in the middle of the quarter, while the AEQ (emotions in class) inventory was completed at the end of the quarter; They were asked to be thinking about a teaching-learning process or specific subject. The data was stored anonymously on a protected server. The researchers only had access to the anonymized responses using a number per participant that allowed the different response files to be linked. Students were invited to participate using an anonymized QR code. After reading the Participant Information Sheet and the Self-Informed Consent Sheet, they were invited to complete different inventories through the INETAS online Platform (www.inetas.net; de la Fuente et al., 2015). They were asked to complete a weekly inventory during a quarter. The SRT-ERT inventory was completed in the middle of the quarter, while the AEQ (emotions in class) inventory was completed at the end of the quarter. They were asked to be thinking about a teaching-learning process or specific subject. The data was stored anonymously on a protected server. The researchers only had access to the anonymized responses using a number per participant that allowed the different response files to be linked. The study was conducted in accordance with the normal ethical principles of the profession of psychology. The data were held in anonymised databases subject to the protections for personal data required by law. The data server is at (NETERRA DATACENTERS EUROPE1). The processing of data was carried out by Mapache Software Europe subject to the appropriate safeguards. The data controller was Project IP1.

#### 2.2.4 Data analysis

An *ex post facto*, prospective, and cross-sectional design was employed (Lohr, 2010). The *ex post facto* approach addresses situations where the variable of interest has already occurred and/or it is unethical to manipulate it. It is termed prospective because the independent variable precedes the dependent variable in the analysis. Additionally, the design is cross-sectional, as longitudinal follow-up is not feasible and data were collected within a short period.

##### 2.2.4.1 Preliminary analysis

The quality of the data was first assessed by identifying outliers and missing cases. Univariate outliers were detected by calculating Z scores for each variable, with Z scores outside the ±3 range considered potentially atypical (Tabachnick et al., 2007). The Mahalanobis distance (D^2^) was used to identify atypical combinations of variables (multivariate outliers), measuring each individual’s multidimensional distance from the centroid of the observations (Lohr, 2010). This helps detect significant deviations from typical variable combinations. Literature suggests either removing or reassigning outliers to the nearest extreme score (Weston and Gore, 2006). This procedure was executed using SPSS (v.26, IBM, Armonk, NY, USA), which includes a specific routine for missing values analysis to determine the extent and nature of missing data (systematic or random).

Assumptions related to sample size, independence of errors, univariate and multivariate normality, linearity, multicollinearity, recursion, and interval measurement level were evaluated, all showing acceptable reliability levels. For sample size, the inclusion of 10–20 cases per parameter is recommended, with at least 200 observations being optimal (Kline, 2005). Independence of errors means that the error term of each endogenous variable should not be correlated with other variables.

Univariate normality was tested by examining the distribution of each observed variable, including indices of asymmetry and kurtosis. Asymmetry values greater than 3 and kurtosis values greater than 10 indicate a need for data transformation. Additionally, Mardia’s multivariate index values below 70 suggest that deviations from multivariate normality are not significant enough to impact the analysis (Mardia, 1970). While the assumption typically requires interval-level measurement, nominal or ordinal variables were sometimes included, provided that their score distributions, especially for dependent variables, were not markedly asymmetric.

Normal distribution of the sample was first confirmed by the Kolmogorov–Smirnov test for dependent variables (Lohr, 2010) and Hoelter’s index was calculated to validate the size of the sample (Tabachnick and Fidell, 2001). Subsequently, descriptive analysis of comparison of means was conducted to test the first and second hypotheses. For purposes of the Hypothesis 3, Pearson’s bivariate correlation was carried out. For the fourth hypothesis, multiple regression analysis was used. For Hypotheses 5, 6 and 7, ANOVA and MANOVA were carried out to confirm the effects among the research variables. Structural equation modelling (SEM) was also carried out using accepted standards of fit. For reliability analysis, Cronbach’s alpha was calculated and specific criteria for direct and indirect effects were applied (Keith, 2019). The programs used were SPSS 26.0 (IBM Corp, 2019) reliability and AMOS v. 23.0 (Arbuckle, 2014) for confirmatory factorial analysis and SEM.

## 3 Results

### 3.1 Descriptive results (hypothesis 1 and hypothesis 2)

In mean analysis, a higher score was obtained for SRT (m = 3.92; SD = 0.79), followed by ERT (m = 3.71; SD. = 0.94). The lowest scores were for DRT (m = 2.64; SD = 0.94) and EDRT (m = 2.54; SD = 1.02) respectively. Scores for SRT (m = 3.92; SD = 0.79) were higher than those for ERT (m = 3.71; SD = 0.94; see Table 1).

**Table 1:** Descriptive statistics for study variables (*n* = 317).

Variable	Mean	DT	Error	Asymmetry	Error	Kurtosis	Error
SRT	3.9156	0.03963	(0.78572)	−0.614	0.123	0.164	0.246
ERT	3.7122	0.04798	(0.94020)	−0.611	0.125	0.118	0.248
NRT	2.7970	0.03669	(0.72834)	0.149	0.123	0.068	0.245
ENRT	2.6881	0.04708	(0.92864)	0.199	0.124	−0.404	0.247
DRT	2.6444	0.04710	(0.93856)	0.136	0.122	−0.554	0.244
EDRT	2.5384	0.05221	(1.02034)	0.210	0.125	−0.698	0.249
PE	3.431	0.687	(0.1686)	−1.95	0.60	0.135	0.120
NE	2.111	0.681	(0.0173)	0.764	0.62	0.458	0.125

SRT = Self-Regulation of Technology; NRT = Internal Non-Regulation of Technology; DRT = Internal Dysregulation of Technology; ERT = External Regulation of Technology; ENRT = External Non-regulation of Technology; EDRT = External Dysregulation of Technology; PE: Positive emotions; NE = Negative Emotions.

In relation to *academic emotions in class* as whole, *positive* academic emotions in class had higher average scores than negative emotions [*t* (316) = 42.109; *p* < 0.001].

### 3.2 Association results (hypothesis 3)

#### 3.2.1 Association of regulatory behavior and context

First, a statistically significant positive correlation was found between NRT and DRT (r = 0.53; *p* < 0.01) and in turn a statistically significant negative correlation was found between DRT and SRT (*r* = −0.13; *p* < 0.01). Conversely, a statistically significant positive correlation was found between ENRT and EDRT (r = 0.59; *p* < 0.01) and in turn a statistically significant negative correlation was found between EDRT and ERT (*r* = −0.14; *p* < 0.01; see Table 2).

**Table 2:** Pearson correlations of internal and external regulation of technology.

	SRT	NRT	DRT	ERT	ENRT	EDRT
SRT						
NRT	−0.126^*^					
DRT	0.171^**^	0.529^**^				
ERT						
ENRT				−0.142^**^		
EDRT				0.172^**^	0.592^**^	

SRT = Self-Regulation of Technology; NRT = Non-Regulation of Technology; DRT = Internal Dysregulation of Technology; ERT = External Regulation of Technology; ENRT = External Non-regulation of Technology; EDRT = External Dysregulation of Technology; ^*^p < 0.05, ^**^p < 0.01.

#### 3.2.2 Regulatory behavior and academic emotions In class

A statistically significant positive correlation was found between SRT and positive emotions (r = 0.34; *p* < 0.01) and in turn a statistically significant negative correlation was found between SRT and negative emotions (SRT; *r* = −0.16; *p* < 0.01). That tendency was similar to a degree for ERT, which had a positive correlation with positive emotions (*r* = 0.30; *p* < 0.01) but had no correlation with negative emotions. In relation to variables of internal and external non-regulation and dysregulation, it was found that NRT and DRT were moderately positively correlated with negative emotions (*r* = 0.40; *p* < 0.01; *r* = 0.34; *p* < 0.01, respectively). It is noteworthy that the emotions most strongly correlated with NRT are anger (0.40) and hopelessness (0.43). Other weak but significant relationships were found between ENR and EDRT and negative emotions (r = 0.34; *p* < 0.01 and r = 0.31; *p* < 0.01), respectively (see Table 3).

**Table 3:** Associations between levels of regulation of technology and emotions in class.

	SRT	NRT	DRT	ERT	ENRT	EDRT
Enjoyment	0.284^**^	−0.117^*^	−0.01	0.252^**^	−0.092	0.033
Hope	0.334^**^	−0.098	−0.009	0.313^**^	−0.047	0.1
Pride	0.310^**^	−0.051	0.048	0.265^**^	−0.03	0.099
Boredom	−0.176^**^	0.351^**^	0.319^**^	−0.126^*^	0.358^**^	0.258^**^
Anger	−0.182^**^	0.402^**^	0.355^**^	−0.120^*^	0.371^**^	0.286^**^
Anxiety	−0.077	0.325^**^	0.255^**^	−0.043	0.192^**^	0.207^**^
Shame	−0.082	0.273^**^	0.211^**^	−0.053	0.167^*^	0.194^**^
Hopelessness	−0.095	0.438^**^	0.369^**^	−0.101	0.363^**^	0.351^**^
Positive	0.343^**^	−0.091	0.017	0.302^**^	−0.067	0.095
Negative	−0.167^**^	0.407^**^	0.346^**^	−0.09	0.349^**^	0.317^**^

SRT = Self-Regulation of Technology; NRT = Non-Regulation of Technology; DRT = Internal Dysregulation of Technology; ERT = External Regulation of Technology; ENRT = External Non-regulation of Technology; Positive E = Positive Emotions; Negative E = Negative Emotions.

* *p* < 0.05,

** *p* < 0.01.

### 3.3 Multiple prediction (hypothesis 4)

#### 3.3.1 External prediction of levels of internal regulation of technology

*Internal Regulation of Technology* (SRT) [*F*(3, 328) = 45,392; *p <* 0,001*; r^2^* = 0.286 (28.60%)], was significantly predicted by ERT (*B* = 0.523, *p < 0.*001). For *Internal Non-Regulation of Technology* (NRT), the external factor model [*F*(3, 328) = 73; *p <* 0,001*; r^2^* = 0.40 (40%)] showed that both ENRT (*F*(328) = 7.5; *p <* 0.001) and EDRT [*F*(330) = 4.99; *p <* 0.001] were statistically significant positive promoters of NRT.

The linear prediction model showed to a degree that was statistically significant [*F*(3, 330) = 125.5; *p < 0*.0.001*; r^2^* = 0.52 (52%)], that *DRT* was predicted by ENR (*B* = 0.232; *p < 0.*001) and EDR (*B* = 0.552; *p < 0*.001).

#### 3.3.2 Emotional predication of levels of internal and external regulation

The linear prediction model tested [*F*(6, 275) = 9,588; *p <* 0.001*; r^2^* = 0.155 (15.50%)] showed that internal (*B* = 0.300; *p* < 0.001) and external (*B* = 0.134; *p* < 0.05) regulation significantly predicted *positive emotion* in class. Conversely the linear prediction model tested [*F*(6, 275) = 10,487; *p <* 0.001*; r^2^* = 0.177 (17.7%)] showed that non-regulatory conduct in relation to ICT (*B* = 0.141; *p <* 0.01) and dysregulatory conduct (*B* = 0.227; *p* < 0.01) of students together with a non-regulatory environment (*B* = 200; *p* < 0.01) significantly predicted *negative emotions* (particularly boredom).

### 3.4 Inferential results (hypotheses 5 and 6)

#### 3.4.1 The effect of the level of external regulation on internal regulation

Analysis of variance (ANOVA) showed a significant effect of type of context on *Total Internal Regulation* (SRTOT) [*F*(2, 316) = 41,815; *p <* 0.001*; r^2^* = 0.444; *Power* = 1], and that ERT significantly determines students’ SRT. Analysis of means identified a group with low total scores for regulation (m = −0.8795; SD = 0.40483; n = 118), a group with moderate level of regulation (m = −0,4,713; SD = 0.040389; n = 124) and a group with high level of regulation (m = 0.0781; SD = 0.42459; n = 74). No significant differences were found in error variance of the groups [*F*(2, 313) = 0.367; *p* = 0.693]. In addition, it was found that levels of external regulation were significantly different in relation to SRTOT [3 < 2 < 1, *p* < 0.001).

#### 3.4.2 The effect of the level of external regulation on academic emotions

There was a significant principal effect of the level of external regulation of ICT on positive and negative emotions [*F*(4,534) = 9,269 (Pillai), *p* < 0.001; *r^2^* = 0.05; power = 1.00], with greater discriminatory power for negative emotions [*F*(2,267) = 19,911 (Pillai), *p* < 0.001; *r^2^* = 0.130; power = 1.00], than positive emotions [*F*(2,267) = 4,481 (Pillai), *p* < 0.001; *r^2^* = 0.035; power = 0.85; see Table 4].

**Table 4:** Mean values of levels of emotion in function of external regulatory context.

	External regulation					
Emotions	1. Low (*n* = 99)	2. Mean (*n* = 118)	3. High (*n* = 53)	Mean (270)		Post
Positive	3.42 (0.69)	3.63 (0.61)	3.74 (0.67)	3.58 (0.67)		1 < 3^**^
Negative	2.37 (0.78)	1.85 (0.58)	1.73 (0.51)	2.06 (0.69)		1 > 2 > 3^**^
Hope	3.50 (0.78)	3.66 (0.67)	3.86 (0.69)	3.64 (0.73)	*F*(2,271) = 4,523^*^	3 > 1^**^
Pride	3.50 (0.73)	3.65 (0.71)	3.74 (0.75)	3.61 (0.73)	*F*(2,271) = 2,084^*^	3 > 2.1^**^
Boredom	2.62 (0.97)	2.03 (0.75)	1.76 (0.78)	2.19 (0.91)	*F*(2,271) = 21,805^**^	1 > 2 > 3^**^
Anger	2.21 (0.86)	2.03 (0.75)	1.49 (0.52)	1.85 (0.75)	*F*(2,271) = 22,381^**^	1 > 2 > 3^**^
Anxiety	2.37 (0.80)	2.13 (0.70)	1.98 (0.67)	2.19 (0.75)	*F*(2,271) = 5,701^*^	1 > 2.3^**^
Shame	2.48 (0.92)	2.15 (0.69)	2.07 (0.72)	2.26 (0.77)	*F*(2,271) = 7,31^*^	1 > 2.3^**^
Hopelessness	2.18 (0.89)	1.70 (0.65)	1.36 (0.41)	1.36 (0.41)	*F*(2,271) = 24,650^**^	1 > 2 > 3^**^

* *p* < 0.01,

** *p* < 0.001.

MANOVA for positive and negative emotions also showed a significant principal effect of the level of external regulation [*F*(14,532) = 5,204 (Pillai), *p* < 0.001; *r^2^* = 0.120; power = 1.00], with significant partial effects. It should be noted that the greatest effect was seen with hopelessness, anger and boredom which showed significant differences in function of each level of teaching context (see Table 4).

#### 3.4.3 Effect of gender on internal and external levels of regulation

MANOVA showed a significant principal effect of *gender* on internal and external regulation [*F*(6, 310) = 3,603*(Pillai)*; *p* < 0.01; *r^2^* = 0.065; *Power* = 0.953]. Pillai’s trace test showed that there were no joint differences between the groups echoing the finding that the matrices of observed covariance of dependent variables are identical across groups (Box mean = 16.207; *p* < 0.135). In turn, significant partial effects were found, with a gender effect on DRT [*F*(1, 311) = 12,044; *p* < 0.001; *r^2^* = 0.037; *Power* = 0.933] and on NRT [*F*(1, 311) = 3,830; *p* < 0.05; *r^2^* = 0.012; *Power* = 0.497]. In both cases, male students scored more highly. Male students also scored significantly more highly for ENRT [*F*(1, 311) = 15,484; *p* < 0.001; *r^2^* = 0.047; *Power* = 0.976] and EDRT [*F*(1, 311) = 6,552; *p* < 0.01; *r^2^* = 0.020; *Power* = 0.723]. Partial effects were found because male students had higher scores for NRT (m = 2.91; SD = 0.80) than female students (m = 2.72; SD = 0.71). Male students also scored more highly (m = 2.88; SD = 0.94) than female students (m = 2.48; SD = 0.91) for DRT.

In relation to *context*, male students had higher scores for ENRT (m = 2.96; SD = 0.93) than female students (m = 2.51; SD = 0.89). For EDRT, the scores of male students (m = 2.70; SD = 1.07) were higher than those of female students (m = 2.37; SD = 0.99; see Table 5).

**Table 5:** Descriptive statistics for study variables (*n* = 317).

	Gender	Mean	St. Deviation
SRT	Female	3.92	0.78
	Male	3.83	0.83
	Total	3.90	0.80
NRT	Female	2.72	0.71
	Male	2.91	0.80
	Total	2.77	0.74
DRT	Female	2.48	0.91
	Male	2.88	0.94
	Total	2.59	0.93
ERT	Female	3.73	0.95
	Male	3.54	1.00
	Total	3.68	0.97
ENRT	Female	2.51	0.89
	Male	2.96	0.93
	Total	2.63	0.92
EDRT	Female	2.37	0.99
	Male	2.70	1.07
	Total	2.46	1.02

#### 3.4.4 The effect of gender on academic emotions

MANOVA identified gender as principal effector for *Positive Emotions* [*F* (1, 461) = 7.959, *p* < 0.01, *η^2^* = 0.017]. However, there were no significant differences with negative emotions [*F*(1, 461) = 0.223, *p* = 0.637, η^2^ = 0.000]. Statistical tests of homogeneity of variance showed that there were no significant differences between variances across groups for both dependent variables (Box m = 5.410; *p* = 0.147). A significant partial effect showed that male students score significantly more highly for rage [*F*(1,472) = 7,854; *p < 0*.001; *r^2^* = 0.016; Power = 0.779; see Table 6].

**Table 6:** Descriptive statistics for academic emotions by gender.

	Sex	Mean	St. Deviation
Enjoyment	Male	3.09	0.71
	Female	3.30	0.70
	Total	3.25	0.70
Hope	Male	3.35	0.75
	Female	3.55	0.69
	Total	3.51	0.71
Pride	Male	3.23	0.68
	Female	3.49	0.71
	Total	3.43	0.71
Boredom	Male	2.50	0.90
	Female	2.24	0.85
	Male	2.30	0.87
Anger	Female	2.07	0.77
	Total	1.81	0.67
	Male	1.86	0.70
Anxiety	Female	2.07	0.76
	Total	2.18	0.69
	Male	2.15	0.71
Shame	Female	2.04	0.79
	Total	2.21	0.87
	Male	2.18	0.86
Hopelessness	Female	1.90	0.74
	Total	1.72	0.65

## 4 Discussion

### 4.1 Discussion of hypotheses and results

The objective of this study was to understand general behavior towards, and use of, ICT in a sample of university students. And to determine the internal and external regulatory factors that predict and differentiate behavior in relation to ICT at university and the relationship of such factors with academic emotions. Additionally, we aimed to study the differentiating effect of gender on these relationships.

In relation to *Hypothesis 1*, it is confirmed in our sample of students that the highest scores obtained were for SRT, followed by ERT. Hypothesis 1 is confirmed inasmuch as the highest scores were obtained for Regulation (SRT and ERT) and the lowest scores were obtained for DRT and EDRT. For *Hypothesis 2*, it is confirmed that SRT scored more highly than ERT.

For *Hypothesis 3*, it was postulated that there is a predictive linear association between the levels of internal and external regulation of technology. More specifically, it was also expected that SRT and ERT would be negatively associated with NRT and ENRT. And that SNR and ENRT would be positively associated with DRT and EDRT. The results obtained support Hypothesis 3. It was found that there is indeed a high positive correlation between DRT and NRT and ENRT and EDRT which suggests that a lack of self-regulation and of a regulatory environment may lead to a higher degree of disorganisation and problems in the use of technology. This result is consistent with the relationship model postulated by *Self- vs External-Regulation Theory* (de la Fuente, 2022, 2024a; de la Fuente et al., 2022a,b) so that this study provides empirical support for that theory in the context of the use of ICT at university, which had not previously been investigated. These results underline the importance of integrating self-regulation of technology into programmes of education and training so as to prepare students to cope with the challenges that they face in a world that is increasingly interconnected and dominated by technology (Díaz-Vicario et al., 2019; Laurence et al., 2020; García-Montero and Bustos-Córdova, 2021).

Conversely, however, we also found a negative relationship between, on the one hand, SRT and ERT and, on the other hand, NRT and ENRT (respectively). This suggests that the higher the level of regulation of technology, internal and external, the lower the number of ambivalent situations propitious to a lack of regulation. It was also found that self-regulated students experience greater academic success, self-efficacy and intrinsic motivation. These findings underline the importance of promoting self-regulation within the teaching process and suggest that that may be key to improved academic achievement and the motivation of students in technological environments (García-Martín, 2012).

*Hypothesis 4* postulated that ERT would promote SRT and have the converse effect on DRT. And that ENRT and EDRT would be positively predictive of DRT. As such and given the results obtained, Hypothesis 4 is borne out. This is because ERT promotes SRT and does not promote DRT. However, it was seen that ENRT and EDRT promote both DRT and NRT. As such, it supports complex interaction between personal and contextual factors and underlines the need to create holistic educational and intervention strategies (de la Fuente et al., 2022c). Dealing with self-regulation of technology does not require us just to consider the dynamics in academia and teamwork, but also to acknowledge the influence of teachers and parents as key facilitators in the inculcation of healthy ICT practice among students (Pan et al., 2020; Pachón-Basallo et al., 2021).

The complementary analysis of the relationship between regulatory variables and academic emotions confirmed our expectations. SRT showed a significant positive correlation with positive emotions and negative correlation with negative emotions. Of particular note was the moderate relationship of NRT with negative emotions, specifically anger and hopelessness. These results are important because they may suggest the nexus between dysregulated use of ICT at university and emotional dysregulation of students. Indeed, it has been shown in a longitudinal study that experiencing anger is directly associated with prosocial conduct and aggression, as measured 2 years later (Mesurado et al., 2018).

The analysis of the *inferential hypotheses* generated significant findings in relation to the influence of variables of internal and external regulation in the university context. It was confirmed that regulation by the environment in which university students find themselves does indeed affect their self-regulation. This shows the critical importance of context in academic performance and the acquisition of competences (Tobar-Otero and Canós-Darós, 2022).

In relation to academic emotions, the results obtained indicate a significant influence of the suite of predictive variables in the SRT vs. ERT model on both positive and negative emotions. There was a particularly notable significant impact of variables of self-regulation (SRT, NRT, DRT) on the prediction of negative emotions. These results provide partial support for the hypothesis postulated since personal variables of regulation showed a considerable influence on the prediction of negative emotions.

In relation to positive emotions, it was determined that SRT, ERT, and EDRT explain approximately 15% of the variability observed in positive emotions, suggesting a significant relationship, albeit less pronounced than with negative emotions. Although some regulatory variables showed significant effects, not all of them had an impact similar to the variables of self-regulation on positive emotions. This finding aligns with previous research indicating that self-regulation is a crucial predictor of well-being and positive emotional outcomes (López-Madrigal et al., 2022). However, the external variables of regulation did not affect positive emotions as clearly as expected, which does not completely support the initial hypothesis. This discrepancy highlights the complexity of the interaction between personal and contextual factors in influencing emotional outcomes.

Moreover, the study by Garzón-Umerenkova et al. (2022) underscores the mediating role of self-regulation in the relationship between academic behaviors and well-being, further emphasizing the importance of self-regulation in promoting positive emotions. Given these findings, future research should explore in greater depth the linear relationships between external regulatory factors and positive emotions, considering additional variables such as coping strategies, resilience, and positivity, as suggested by the 3P Model (Biggs, 1989). This approach could provide a more comprehensive understanding of how educational contexts and personal regulatory capacities jointly influence emotional well-being in university students.

In addition, it is crucial to deepen our understanding of how variables of regulation in relation to ICT can favour or disfavour emotional regulation and experience of students. It is recognised that emotional regulation is a determinative variable that appears to play an important role in the development of negative affect and psychosomatic symptoms in students in higher education (Teixeira et al., 2022). Such additional information would be essential to better understanding the impact of personal regulatory variables in the academic and emotional contexts of university students.

The *analysis of gender differences* showed differences for SRT and ERT. Male students scored more highly for DRT and EDRT, which is indicative of habits that are deficient in terms of self-regulation and exposure to disruptive environments. Prior research has underlined the differentiating, predictive role of gender and age in learning behavior. Self-regulation, as a key personal characteristic, reaffirms the importance of considering such factors when designing educational strategies and interventions to improve university learning (de la Fuente and Martínez-Vicente, 2023a,b).

Finally, and in relation to academic emotions, it was observed that female students experience slightly more positive emotions that males, with a statistically significant difference, albeit of reduced magnitude. However, no notable differences were found in negative emotions between the genders. It should be noted that the specialist literature has detected gender differences and shown that women tend to score more highly for emotions such as enjoyment, hope and pride relative to men (Barron and Harackiewicz, 2001; Pekrun et al., 2004, 2007).

#### 4.1.1 Limitations

This study has limitations in terms of geography and demographics, as it considered only university students in Spain. Although the students in the sample were studying a number of disciplines, this concentration restricts the scope for generalizing the results to other populations. Additionally, the number of participants, although significant, may limit the extrapolation of the findings, particularly given the predominance of women in the sample. These limitations highlight the need to interpret the results in light of the specific context of the sample, acknowledging that differences from other populations and areas of study may affect the applicability of the conclusions. Furthermore, the cross-sectional design of the study limits the ability to infer causality between the variables studied. Future research should consider longitudinal designs to better understand the causal relationships and the long-term effects of ICT regulation on academic and emotional outcomes.

#### 4.1.2 Future research

It would be valuable for future research to analyse in greater depth how specific contexts, such as university policies and access to technology resources, impact regulation of ICT. It would also be valuable to explore in greater detail how the interactions between and among students, teaching staff and the academic environment may influence the regulation of technology. As well as investigating gender differences in self-regulation, it would be important to understand the reasons behind such differences. It would also be of value to research the relationship between academic emotions and the appropriate use of ICT and to explore possible linear relationships.

### 4.2 Implications of the results for the practice educational psychology

#### 4.2.1 Training of university teaching staff and educational context

Another implication of this study is the need to provide training to teaching staff so that they can promote self-regulation and appropriate use of ICT by students. Teaching staff can play a key role in the creation of educational environments that promote positive regulation in relation to technology. That could include teaching strategies that help students to develop skills of self-regulation and the adoption of university polices that promote the healthy use of ICT. As such, it is essential that both teaching staff and universities seek ways to limit negative aspects of ICT whilst exploiting it to the full. That could include awareness-raising programmes and the promotion of best practice in the use of technology at university, working with educational psychologists.

#### 4.2.2 Model of interactive evaluation and intervention

Given that this study suggests a complex relationship between self-regulation and external regulation, it is crucial to adopt an interactive model of evaluation and intervention. That implies that intervention strategies should take account of both internal and external regulatory factors. That may require collaboration between psychologists, teaching staff and other people working in education to effectively address the challenges related to the use of ICT that arise at university.

In summary, these findings underline the importance of addressing regulation of technology in a university context and highlight the need to develop intervention strategies to promote self-regulation, promote co-regulation among students and to ensure appropriate use of new technology. In synthesis, the study brings into relief the importance of promoting the responsible and ethical use of ICT in education. That entails raising the awareness of young people, the training of teaching staff and guidance to parents and schoolteachers in addressing the challenges of the digital world (Prats-Fernández et al., 2018; Gómez-Vahos et al., 2019).

#### 4.2.3 Programmes of work to prevent and mitigate the problem

Interventions based on developing psychological resources have had positive impacts on daily life and wellbeing. Studies such as the RedSocs educational workshops, intended to promote the healthy use of technology among 1,200 students, parents and educators in Catalonia, have demonstrated their efficacy in promoting the appropriate use of the Internet (Prats-Fernández et al., 2018). In the same way, the Cubilete programme conducted in students in Jaen successfully reduced excessive Internet use and negative behaviors in relation to online gaming and betting (Berrios Aguayo et al., 2020). Recently, the Positive Emotional Training (PoET) programme has been trialled successfully in the general population and shown a significant reduction in symptoms associated with depression and anxiety together with a notable increase in optimism through online sessions (Niemann et al., 2023).

Further, the study by Rincón et al. (2023) focused on the evaluation of the effectiveness of the mobile app OneUS in promoting positive emotions and positive thinking so as to improve general wellbeing. That study represents a significant advance in the development of an evidence-based application focused on intentional mental training so as to promote wellbeing. Despite that, abandonment by participants and the limited generalisability of the results to clinical populations were acknowledged as challenges for the study.

## 5 Conclusion

The results of this investigation have revealed that university students should increase their competence in the use of ICT. It was also possible to observe a positive correlation between self-regulation and external regulation, which suggests that the teaching context in which students find themselves plays a crucial role in the way in which students manage their technology-related behavior. We also identified that greater self-regulation is associated with lesser dysregulation. The results of this study highlight the importance of seeking strategies to promote appropriate use of new technology within universities among students and educators.

It is fundamental that practising psychologists recognise the importance of regulation of technology and are prepared to address issues around the use of ICT. In that sense, it would be possible to develop a formative *model of competence* based on recent models (de la Fuente and Martínez-Vicente, 2023a,b) that includes strategies for intervention aimed at both students and at teaching staff. The promotion of self-regulation through digital education and awareness-raising in relation to the risks of inappropriate use of ICT.

It is also essential to bear gender differences in mind because male students scored more highly for Dysregulation of Technology and showed greater exposure to contexts that are dysregulatory in relation to technology. It is essential to develop specific intervention strategies that address those differences and promote co-regulation among students.

## Data availability statement

The raw data supporting the conclusions of this article will be made available by the authors, without undue reservation.

## Ethics statement

The studies involving humans were approved by Research Ethics Committee of the University of Navarra (Ref. 2018.170). The studies were conducted in accordance with the local legislation and institutional requirements. The participants provided their written informed consent to participate in this study.

## Author contributions

JF: Conceptualization, Funding acquisition, Project administration, Writing – original draft, Writing – review & editing. LL-L: Data curation, Formal analysis, Writing – original draft. MP-B: Resources, Software, Writing – original draft, Writing – review & editing. LM-Í: Formal analysis, Methodology, Writing – original draft. PB-S: Conceptualization, Writing – review & editing.

## Footnotes

^1^www.inetas.net
